# Intermediate tree cover can maximize groundwater recharge in the seasonally dry tropics

**DOI:** 10.1038/srep21930

**Published:** 2016-02-24

**Authors:** U. Ilstedt, A. Bargués Tobella, H. R. Bazié, J. Bayala, E. Verbeeten, G. Nyberg, J. Sanou, L. Benegas, D. Murdiyarso, H. Laudon, D. Sheil, A. Malmer

**Affiliations:** 1 Swedish University of Agricultural Sciences (SLU), Department of Forest Ecology and Management, SE90183 Umeå, Sweden; 2 Institut de l’Environnement et de Recherches Agricoles, Département Productions Forestières, 03 BP 7047 Ouagadougou 03, Burkina Faso; 3 Université de Ouagadougou, Unité de Formation et Recherche en Sciences de la Vie et de la Terre–03 BP 7021 Ouagadougou 03, Burkina Faso; 4 World Agroforestry Centre (ICRAF), West and Central Africa Regional Office, Sahel Node, BP E5118, Bamako, Mali; 5 Institute for Biodiversity and Ecosystem Dynamics (IBED), University of Amsterdam, P.O. Box 94240, 1090 GE Amsterdam, The Netherlands; 6 Tropical Agricultural Research and Higher Education Center (CATIE). Headquarters 7170, Cartago, Turrialba 30501, Costa Rica; 7 Department of Geophysics and Meteorology, Bogor Agricultural University, Jl. Meranti, Kampus Darmaga, Bogor, 16115, Indonesia; 8 Center for International Forestry Research, Jl. CIFOR, Situgede, Bogor 16115, Indonesia; 9 Department of Ecology and Natural Resource Management (INA), Norwegian University of Life Sciences (NMBU), Box 5003, 1432 Ås, Norway; 10 School of Environmental Science and Management, Southern Cross University, P.O. Box 157 Lismore, NSW 2480, Australia; 11 Department of Business Administration, Technology and Social Sciences Luleå University of Technology, SE-97187, Luleå, Sweden

## Abstract

Water scarcity contributes to the poverty of around one-third of the world’s people. Despite many benefits, tree planting in dry regions is often discouraged by concerns that trees reduce water availability. Yet relevant studies from the tropics are scarce, and the impacts of intermediate tree cover remain unexplored. We developed and tested an *optimum tree cover theory* in which groundwater recharge is maximized at an intermediate tree density. Below this optimal tree density the benefits from any additional trees on water percolation exceed their extra water use, leading to increased groundwater recharge, while above the optimum the opposite occurs. Our results, based on groundwater budgets calibrated with measurements of drainage and transpiration in a cultivated woodland in West Africa, demonstrate that groundwater recharge was maximised at intermediate tree densities. In contrast to the prevailing view, we therefore find that moderate tree cover can increase groundwater recharge, and that tree planting and various tree management options can improve groundwater resources. We evaluate the necessary conditions for these results to hold and suggest that they are likely to be common in the seasonally dry tropics, offering potential for widespread tree establishment and increased benefits for hundreds of millions of people.

Two-thirds of the world’s population may live in water-limited regions by 2025^1^. In Africa about 340 million people already lack access to adequate and hygienic sources of water, such as groundwater^2^. Limited water constrains food production, nutrition, and health as well as impacting opportunities for education, work, and improved livelihoods. Reliable access to clean water is essential for achieving the UN Sustainable Development Goals.

Forests have often been described as ‘sponges’ storing rain water and slowly releasing it to maintain groundwater and streams during dry periods^3^,^4^,^5^. Formerly, this *sponge theory* and related ideas motivated policies aimed at conserving and restoring forests^5^,^6^,^7^. In recent decades, however, these ideas have lost credibility as studies show that forest clearance generally leads to increased and afforestation to reduced water yields^3^,^8^,^9^. Therefore a contrasting *trade-off theory*–in which more trees means less water–has become the dominant paradigm. This *trade-off theory* predicts that as tree densities increase, water losses from transpiration and interception dominate their hydrological effects^6^,^8^,^10^.

In the tropics the *trade-off theory* rests on limited evidence. The few available studies compare extremes: open land versus closed forest, or grasslands versus dense plantations^11^. Despite the recognition that trees can improve soil hydraulic conductivity and reduce overland water flow^4^,^12^,^13^, and other findings that question the generality of the *trade-off theory*^14^,^15^,^16^, we find no available data on the relationship between tree cover and water yields at intermediate tree densities, and few data concerning the specific mechanisms that determine groundwater reserves and dry season stream flows^11^,^12^,^17^. The neglect of intermediate tree cover is a striking omission given the importance of such open vegetation in terms of extent and biodiversity^18^, and the fact that it supports many of the world’s poorest people. In Africa, there are 350 Mha of open and fragmented forests and 514 Mha of other wooded lands (including savannah, agroforests etc.)^19^–more than the area under closed forest and plantations (277 and 8 Mha, respectively). Such open vegetation also plays a major role in the global carbon balance; regions with 10 to 30% tree canopy cover are estimated to store 23% of the total forest biomass carbon stock (above- and belowground) in sub Saharan Africa and 15% of the total forest carbon stocks for the global tropics^20^. Tree planting is, or would be, a major element in many climate mitigation projects, in efforts to combat desertification, and in livelihood focused development proposals seeking to improve access to firewood and other products. But the *trade-off theory* has reduced the application of such planting projects due to concerns that these efforts would jeopardize scarce water resources^6^,^8^,^9^,^10^,^21^,^22^.

Here we present and test an *optimum tree cover theory* for groundwater recharge that can reconcile the available scientific evidence and contrasting perceptions about forests and groundwater in the seasonally dry tropics (Fig. 1). We hypothesized that under conditions typical of the seasonally dry tropics an intermediate tree cover maximizes groundwater recharge. Below this optimum cover, the hydrological benefits gained from more trees outweigh their extra water use, while at higher values of tree cover the water use from additional trees exceeds any positive effect they might have on groundwater recharge (Fig. 1). We recognize that the tree cover value where this optimum occurs depends on various factors including tree species, local soil and climatic conditions. But before considering these influences we needed to evaluate our theory. We chose a common African semi-arid landscape known as “agroforestry parkland” where water shortage is a recognised livelihood constraint^23^. Parklands, in which annual crops are grown under scattered trees, constitute the most extensive farming system in semi-arid West Africa^23^. Indeed, about 1.9 million km^2^ (47%) of the total agricultural land in Sub-Saharan Africa has a tree cover above 10%^24^. We want to know how such intermediate tree cover influences groundwater recharge.

## Results

**Figure 2:**
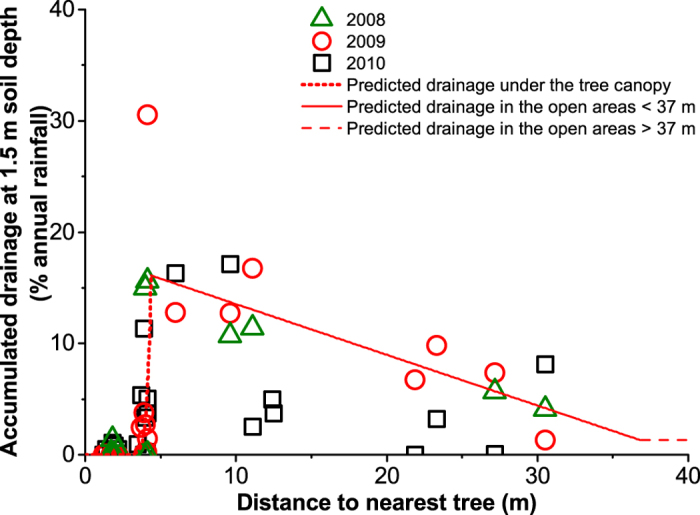
Relationship between accumulated water drainage at 1.5 m soil depth and distance to nearest tree. Accumulated drainage in open areas decreases with the distance from canopy edges in an agroforestry parkland, Burkina Faso. The lines show the relationship used for spatial simulations which was fitted by least squares regression to the 2009 data (see Methods section for details). The red solid line depicts the linear relationship found in the open areas (y = 18.1 − 0.46x; r^2^_adj_ = 0.69, p = 0.013), while the red dotted line depicts the exponential relationship found under the tree canopy (y = 4.3 10^−8^ e^4.5x^; Lack of Fit test indicated a suitable model; p = 0.983). The dashed line is the constant value for the accumulated drainage corresponding to 1.3% of the annual rainfall assumed for the distance range above 37 m. The number of observations was 18, 22 and 25 in 2008, 2009 and 2010, respectively. In 2008, 2009 and 2010 the rainfall during the sampling period represented 85, 99 and 60% of the annual rainfall, respectively.

Our results confirm that groundwater recharge can be maximized at a specific, non-zero, tree cover. We used field measurements of subsurface drainage and tree transpiration to model groundwater recharge in a water budget model related to tree cover and its spatial distribution (see Methods section). Field data from wick lysimeters revealed that the percentage of the yearly rainfall percolating to 1.5 m soil depth reached its maximum of 16% of the annual rainfall around the edge of the tree canopy, 4.4 m from the nearest tree stem, and decreased to 1.3% in open areas, 37 m away from the nearest tree (Fig. 2). This decrease in drainage with increasing distance from the canopy edge followed a linear relationship (y = 18.1 − 0.46x(SE 0.12); where y is the drainage at 1.5 m soil depth and x the distance to the nearest tree stem; r^2^_adj_ = 0.69; p = 0.013). For distances beyond 37 m we assumed that the drainage was 1.3%, which corresponds to the lowest observed value. Therefore, less water is available for groundwater recharge in locations further from any tree and resulting in negligible groundwater recharge when trees are absent.

**Figure 3:**
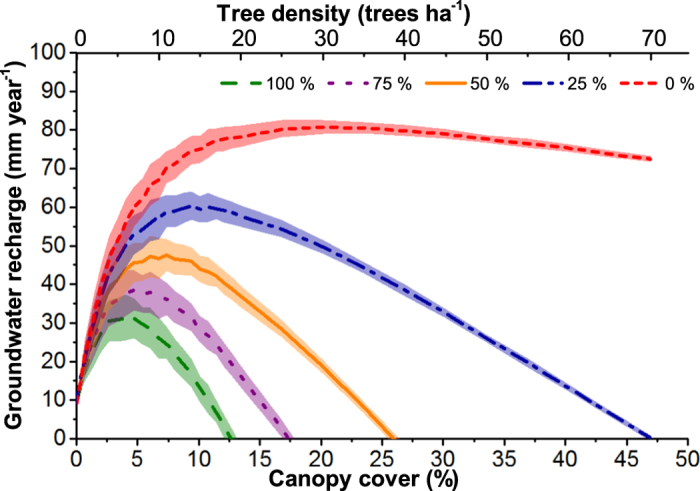
Spatial simulations of groundwater recharge in relation to tree density and canopy cover. The simulations are based on sap flow measurements and on the observed relationship between drainage below 1.5 m soil depth in 2009 and distance to the nearest tree in an agroforestry parkland, Burkina Faso. For each tree density the averages and standard deviations resulting from 100 simulations with random locations of trees on a 1 ha area are shown. The effect of the proportion (0%, 25%, 50%, 75%, 100%) of tree water uptake below 1.5 m is demonstrated by the different colored lines. Average tree size (67 m^2^ canopy area) is assumed for all the simulations.

We remain uncertain concerning the depth from which trees access soil water, and how such water use patterns vary in space when there are multiple trees present: to address these unknowns we modelled groundwater recharge using different scenarios. The results show that in the scenario where 50% of tree water use occurs deeper than 1.5 m in the soil, recharge increases from 9 to 20 mm year^−1^ when tree density increases from zero to just one tree ha^−1^ and reaches 47 mm year^−1^ at 10 trees ha^−1^ (Fig. 3). Even if all transpired water was drawn from deeper than 1.5 m an optimum recharge of 30 mm year^−1^, a value three-times the treeless scenario, occurs at 5–10 trees ha^−1^ density. Beyond the optimum tree cover, recharge decreased but remained higher than in scenarios without trees by up to 10 and 20% canopy cover in the 100% and 50% uptake scenarios, respectively. While the choice of scenarios influenced the values of the optimum tree cover, none questioned the more general conclusion that such an optimum cover existed (more than 0% and less than 100%).

**Figure 4:**
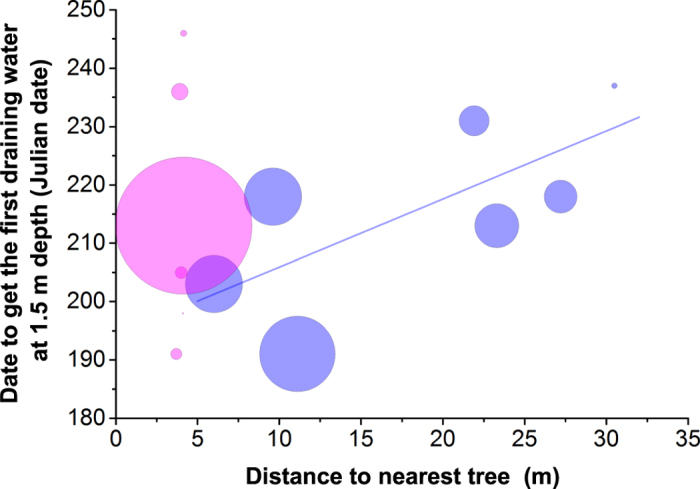
Scatter plot of the date when the first water was collected at each lysimeter versus the distance to the nearest tree (for 2009). The pink and blue circles show lysimeters located under tree canopies and in open areas respectively. The size of the circles is proportional to the yearly accumulated water drainage at 1.5 m soil depth. The solid line shows the linear relationship found in the open areas (y = 194.2 + 1.2x, r^2^_adj_ = 0.40, p = 0.075).

Our measurements show that in the open areas, there was a significant relationship between the yearly accumulated drainage at 1.5 m soil depth and the date in which the lysimeters recorded the first water following the beginning of the rainy season (y = 73.5 − 0.3x(SE 0.06); where y is the drainage at 1.5 m soil depth and x the date to get the first draining water; r^2^_adj_ = 0.81; p = 0.004), while under trees there was no trend. Moreover, in the open areas, the date at which the first draining water was recorded was positively related to the distance to the nearest tree (y = 194.2 + 1.2x(SE 0.52); where y is the date to get the first draining water at 1.5 m soil depth and x is the distance to the nearest tree stem; r^2^_adj_ = 0.40; p = 0.075), with open areas closest to trees receiving the first draining water about 30 days earlier on average than those located furthest away from trees (Fig. 4). In summary, open areas close to trees not only had a higher yearly accumulated drainage at 1.5 m soil depth compared to those further away from trees, but they received the first wet season water earlier.

## Discussion

How can we explain these results? Soil infiltration is improved by trees through litter inputs and roots, promoting higher activity of soil animals. This results in increased soil hydraulic conductivity due to enhanced organic matter content, topsoil aggregation and macro porosity^25^,^26^. The likely cause for the observed difference in amounts and time of water to reach deep soils, as indicated by a previous study in the same area, is that there is not only less water infiltrating in areas far from canopy edges, but water also percolates through the soil more slowly because of fewer macro pores^27^. Open areas are also more exposed to sunshine and heat and thus to losses from evaporation. For example, δ^18^O values in topsoil water in a *Faidherbia albida* parkland in Burkina Faso have been shown to be greater in open areas rather than closer to trees, indicating that evaporation is reduced near to tree cover^28^. The net effect is that rain water falling near to any tree is more likely to percolate to the subsoil than is rain falling further away, which is more likely to evaporate. In our study area, the depth of the water table is well below 2 m during most of the year including the rainy season. Therefore, capillary rise was not considered. If, in other sites where the water table was closer to the ground surface, capillary rise did occur, the resulting rate of loss to evaporation would be lower closer to trees, where there is more macroporosity and less evaporation than in more open areas. Thus, our conclusions about the benefits of tree cover are likely to apply to such cases too. While tree cover improves drainage in the zone outside the canopy, no percolation was detected under the canopy–presumably due to interception and transpiration. Tree canopies may also redistribute rainfall through canopy drip at the outer edge of the canopy, which would also explain the increase in water drainage in the area beneath the canopy edge. The influence of this on the overall drainage patters observed beyond the canopy edge is likely to be relatively minor because, if soil infiltration properties were homogeneous, any such larger scale influence would be symmetrical.

Given the importance of improved water reserves and the various livelihood and environmental benefits that may be gained from greater tree cover, it will be crucial to replicate our results and characterise their applicability to other sites and regions. We are optimistic that optimal recharge will occur at intermediate tree densities over large areas. This optimism is based on consideration of a requirement of our model. The gains from improved infiltration and reduced soil evaporation due to an individual tree must surpass the water used by that tree. When rain intensity exceeds soil infiltrability overland flow occurs and improved infiltrability can increase groundwater recharge. Rain in the seasonally dry tropics is often intense–in the Sahel half of the total annual rainfall amount falls at intensities above 20 to 50 mm h^−1  ^29,30– while soil infiltrability without trees is typically lower^31^,^32^,^33^. The result is that overland flow is common in much of the seasonally dry tropics^32^. The soil in our study site is an Alfisol: these soils are characterized by unstable aggregation, sensitivity to crusting and low infiltrability–characteristics which encourage overland flow^32^. Alfisols comprise some seven million km^2^ in the semi-arid tropics^34^, while related soil types with similar physical constraints cover another eight million km^2 ^34,35. Combining these figures suggests that without trees overland flows would be commonplace over at least 71% of the semi-arid tropics.

We know less about the net hydraulic benefits due to individual trees. In our study area median infiltrability values increased from below 10 mm h^−1^ to 40 mm h^−1^ in open areas among trees and under trees respectively^27^. Under a natural forest about 130 km from our study area and within the same climatic zone, infiltrability values are generally higher than those under trees in our more modified parkland system^36^. A recent meta-analysis of soil infiltrability changes due to tree planting in afforestation and agroforestry experiments found 15 relevant cases involving a total of 10 different species (with age from 1 to 12 years). Of these 11 involved Alfisols and 4 Ultisols. All plantings resulted in increased infiltration ranging from 1.6 to 9 times improvement (with the lowest values for the youngest plantings)^33^. We know that the positive effects of trees on soil infiltrabilty occur across a wide range of conditions, such as with and without tillage^27^,^33^.

We note that the tree species in our study have comparably high water consumption. This suggests that, as long as gains in soil hydraulic properties are similar among species water use versus gains in soil hydraulic properties will be conservative compared to many other tree species in the seasonally dry tropics (see Supplementary Fig. S1 for a comparison of daily water use with other semi-arid trees).

Naturally patterned (i.e. clumped and banded) tree cover is widespread across arid and semi-arid regions of the tropics^37^. Ecologists have sought an explanation for the formation of such patterns. Our study, like various others, indicates that trees exert spatially distinct positive and negative influences on soil water availability and hydraulic properties (see also^38^,^39^). The main positive influence of trees on the soil water budget is via soil infiltrability. Local differences in infiltrabilty can lead to the redistribution of surface runoff to areas under trees, as observed in other dryland vegetation systems^39^. Runoff and runon processes can in turn increase net water stored in the soil close to trees, reinforcing vegetation growth and establishment^39^. The different scales of the positive and negative feedbacks between plant density, soil infiltrability and water use has been suggested as a mechanism explaining banded and clumped vegetation patterns in many semi-arid areas^40^,^41^. We speculate that the distinct positive and negative spatial factors determine the tree patterns found in many natural savannas^37^,^38^– while such patterns require further evaluation they also suggest that the necessary hydrological influences including the improved groundwater recharge near to trees are widespread phenomena.

We acknowledge that in specific circumstances benefits from trees may be reduced or absent. In some regions rain intensities may remain too low or soils too porous for overland flow to occur. In these cases, the overall effect of increased tree cover on groundwater recharge will primarily be determined by the balance between their increased water use and their effect on reducing soil evaporation, and therefore the resulting impact will likely be small or negative. For example, 1% of the semi-arid regions occur over porous soils on volcanic bedrock that have such high inherent infiltration capacities that rates exceed typical rain intensities so that run-off is negligible^42^: in such contexts even scattered trees are unlikely to improve groundwater recharge. Also, there may be circumstances when rainfall amount or intensity is too low for trees to make much difference. Trees may not improve groundwater recharge where removal of litter, grazing and fuelwood harvesting prevent the inputs of organic material required to improve soil infiltration^43^.

Our conceptual model implies that the optimum tree cover for groundwater recharge will be influenced by many factors including several under management control. Tree spatial distribution, for example, can have an important impact as shown by the results from our model for a regular spaced distribution (Supplementary Fig. S2). At the optimum canopy cover the simulations with the largest tree to tree distance achieved 35% higher groundwater recharge than the average random distribution. Therefore, a relatively regular well-spaced tree distribution may be preferable to a random or aggregated one. Tree size, age and species that affect transpiration will also have an influence. We explored the effect of tree size based on tree size transpiration relationship. The groundwater recharge at optimum tree cover increased from 36 to 55 mm when average canopy area for an individual tree was reduced from 130 to 40 m^2^ (Supplementary Fig. S3). Though larger trees yielded a lower optimum than smaller trees, it was less sharply peaked leading to a greater beneficial influence of trees at higher total canopy cover. In our study area the dominant tree species was capable of maintaining high transpiration levels through the dry season. Trees with different water-use strategies–for example deciduous species, which shed their leaves during the dry season–may provide further enhancement in groundwater recharge. Many other plant characteristics including those related to root architecture, shade and litter are also likely to be influential. Further study is required to clarify improvements through the selection of tree species and varieties. We also see opportunities for assessing and defining the influence of tree densities and configurations, and of tree thinning and pruning. In our study area, and elsewhere in Africa, crown pruning is common and is estimated to decrease water losses from transpiration by over 75%^44^. A rough estimate suggests that such pruning could mean that tree cover remains positive for groundwater recharge up to as many as 60 trees ha^−1^ instead of 16 trees ha^−1^ (40 and 11% canopy cover before pruning) even under the conservative assumption that all water used by trees comes from below 1.5 m. Benefits in groundwater recharge may also be achieved through management of livestock grazing. While high stocking rates will typically lead to reduced soil infiltrability as a result of soil compaction, reduced vegetation cover and reduced topsoil organic matter, there may be cases where some grazing can improve soil infiltrability due to manure inputs and soil crust fragmentation^36^,^45^,^46^. In addition to the different management scenarios, we also considered various other assumptions and potential sources of error (see Supplementary Discussion for details). None undermine our main conclusion, i.e., that optimal groundwater recharge will often result from intermediate tree cover.

In our site in Burkina Faso, with annual rainfall ranging from 570 to 1180 mm, the differences in groundwater recharge indicated by our simulations (30–80 mm year^−1^) are substantial being 2–14% of the total gross water input. Further improvements are possible through management choices. Differences of these magnitudes can translate into improved water availability and agriculture production for millions of people in water limited regions. We hope that our results will stimulate research on the hydrological impact of tree cover in a wider range of systems including agroforests, selectively harvested and old growth forests, savannah woodlands and pastoral systems. We envisage significant opportunities to improve land-use policies, practices and management interventions. One recent summary suggests that close to 1.5 billion ha of land in the tropics offer opportunities for the restoration of, or increase in, tree cover^47^ offering multiple goods and services to local people and a number of global benefits such as improved carbon sequestration^20^ and biodiversity conservation^18^. Our results suggest that intermediate tree cover might–if managed judiciously–improve water availability over vast areas benefitting hundreds of millions of people.

## Methods

### Site description

**Figure 5:**
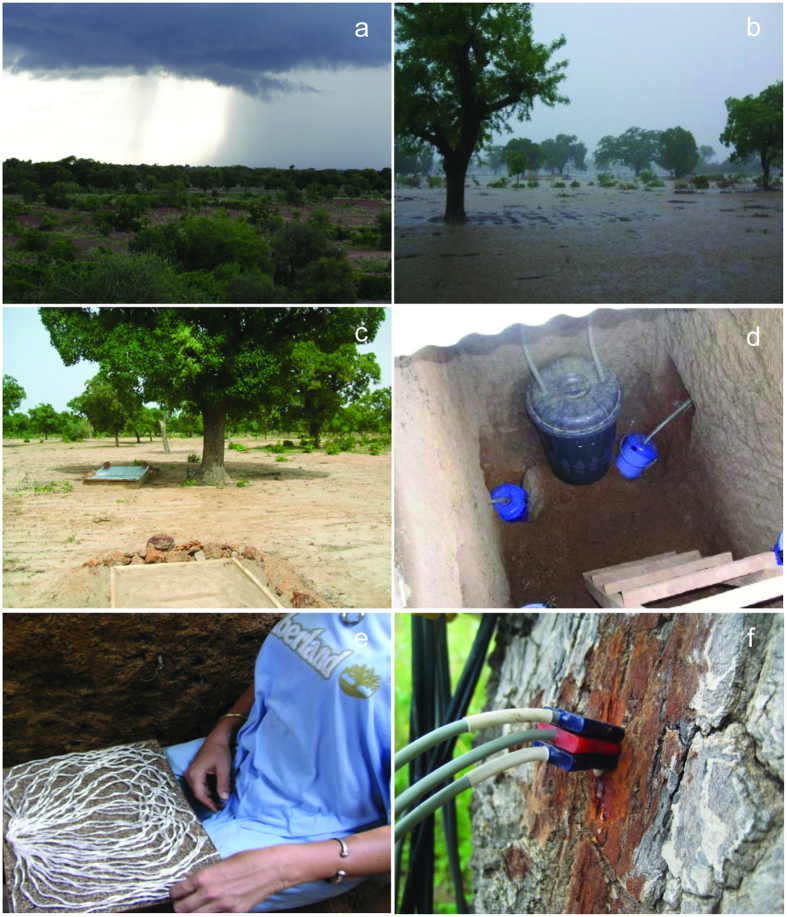
Saponé study area, Burkina Faso, and installations. **(a)** Overview of the landscape from an elevated position. **(b)** Overland water flow during rainfall among the Shea trees (*Vitellaria paradoxa*) in the cultivated landscape. **(c)** Soil pits (see rectangular structures) under the canopy of a Shea tree (behind) and in the centre of an open area (front). **(d)** Lysimeter instalations on opposite sides of a soil pit at 1.5 m soil depth. One lysimeter was located towards the tree 1 to 2 m from the trunk; the second on the far side at 4 to 5 m from the trunk. **(e)** Passive capillary fiberglass wick lysimeter prior to installation. **(f)** HRM30 heat ratio probe for sap flow measurements attached to the stem of a Shea tree.

**Figure 6:**
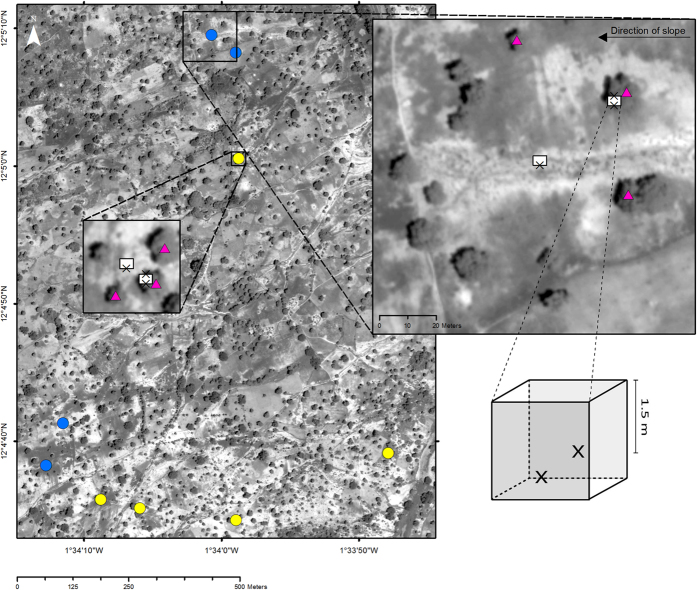
Overview of the study area and sampling design. Satellite image (Panchromatic WorldView-2 image from 18 July 2012) of the study area showing the 9 sampling locations, 4 corresponding to large open areas (blue dots) and 5 corresponding to small open areas (yellow dots). Enlargements of a large and a small open area are also shown. The soil pits were located in each sampling location both at the centre of the open area and under a tree (white boxes). Soil water drainage was collected with lysimeters located at 1.5 m soil depth at three points in each sampling location (X). Sap flow was measured in three trees per sampling location (pink triangles).

The study was conducted in an open woodland managed for crops, grazing, fuel wood and non-timber forest products in Saponé (Fig. 5a,b), a village located 30 km south of Ouagadougou, Burkina Faso, West Africa (12°04′48″N, 1°34′00″W, Fig. 6). These systems are regionally referred to as “agroforestry parklands”^48^: they are the dominant farming system in semi-arid West Africa and resemble tree based systems common in other parts of the world^23^. Parklands originate from the clearing of natural woodlands for cropping and farmer selection and protection of given tree species^23^. Tree density usually ranges from 5 to 100 trees ha^−1 ^23.

The study site is located on a peneplain at 310 to 325 m above sea level. Mean annual temperature (1952–2008) at Ouagadougou (the nearest meteorological station) is 28 °C with a mean annual precipitation of 787 mm year^−1^ (1952–2010), ranging between 570 and 1180 mm year^−1^ (Direction de la Météorologie du Burkina Faso). The single rainy season generally starts around April and ends in October. About 80% of the average annual precipitation falls between June and September (Supplementary Fig. S4). Mean annual potential evapotranspiration (PET) is 1900 mm year^−1^ (1974–2003), ranging between 1200 and 2100 mm year^−1^. The soils of the area have been classified as *sols ferrugineux tropicaux lessivés*^49^, corresponding to Ferric Lixisols^50^. They possess sandy clay textures and low nutrient content (N = 0.03%, Olsen extractable P = 1.05 ppm and exchangeable bases <2.5 cMolc kg^−1^)^51^.

The dominant tree species of the agroforestry parklands in and around Saponé is *Vitellaria paradoxa* C.F.Gaertn. *Sapotaceae* (*Karité* in French and *Shea* in English), which is considered the predominant parkland tree species in semi-arid West Africa^52^. Other tree species present in the parkland are *Parkia biglobosa* (Jacq.) G.Don, *Azadirachta indica* A.Juss*., Diospyros mespiliformis* Hochst. ex A.DC.*, Adansonia digitata* L.*, Acacia nilotica* (L.) Delile*, Ficus gnaphalocarpa* (Miq.) Steud. ex Miq.*, Khaya senegalensis* (Desv.) A. Juss.*, Lannea microcarpa* Engl. & K. Krause*, Sclerocarya birrea* (A. Rich.) Hochst.*, Tamarindus indica* L. and *Terminalia laxiflora* Engl. The tree density in the study area, which comprises ca. 100 ha, averages 21.3 trees ha^−1^ (SD = 16.3; n = 20) but ranges between 4 and 62 trees ha^−1^, while canopy cover ranges between 3 and 21% (SD = 5; n = 20) (estimated on 20 100 × 50 m plots on satellite images from year 2002 (source: Google Earth 2012; Digital Globe 2012). In the study area Shea trees have an average DBH (diameter at 1.3 m height) of 45 cm (SD = 17; n = 67) and an average ground projected canopy area, estimated from canopy diameter, of 67 m^2^ (SD = 39; n = 67). The average canopy area in our study area (67 m^2^) is close to the average for *Vitellaria paradoxa* based on data from Mali, Burkina Faso and Cameroon (54 m^2^)^52^. The trees are scattered forming an open woodland which is often associated with diverse cultivated plots of annual crops such as millet, sorghum, cowpea and beans^41^. These plots are regularly fallowed for 3–5 years. Trees are kept in the agricultural fields for multiple reasons - including fruits, shading and fodder for animals. Livestock is present in the area, mainly moving on tracks and in the fallows, but is kept out of the agricultural fields during the cropping season and allowed to graze crop residues after harvest.

Shea trees are generally considered deciduous but in the study area the trees are rarely leafless because they replace their leaves progressively^53^. Its lateral roots mostly follow the ground surface and can extend 20 m from the tree and reach a depth of 0.4 m^54^,^55^.

### Sampling design

The sampling was designed to estimate changes in groundwater recharge as a function of tree proximity and density. Two classes of areas were selected to span a range of tree densities: large open areas (large canopy free areas, having a radius between 22 and 30 m) and small open areas (radius between 6 and 13 m). The sampling locations were selected according to the following criteria: 1) The radius of the open area had to be either below 15 m or above 20 m. Open areas with intermediate sizes were not considered; 2) All trees bordering the open area had to be Shea trees; and 3) Sampling locations had to be present both North and South of a settlement located at the center of the study area. Each sampling location contained three measurement points where soil water drainage was collected at 1.5 m soil depth (Fig. 6). For information about replicates see Table 1. One measurement point was located in a soil pit in the centre of each open area selected. The other two were located in opposite sides of a second pit close to a Shea tree (Figs 5c and 6) with one of the measurement points at a distance between 1 and 2 meters from the stem and the other in the opposite side of the pit towards the canopy edge, 4–5 meters from the stem. The soil pits were orientated with the long side, where the lysimeters are located, in the slope direction to minimize disturbance by lateral water flow (Fig. 6).

Soil water drainage was collected at 1.5 m soil depth daily during the rainy season in 2008, 2009 and 2010 in 6, 8 and 9 sampling locations respectively (Table 1), corresponding to 18, 22 and 25 measurement points. At the end of the rainy season in 2008 one pit next to a tree was destroyed due to heavy rains. These two measurement points could not be included in the following years. Twenty-seven healthy and not recently pruned Shea trees were selected for sap flow measurements. This involved three trees per sampling location over the 2008–2010 period. In each sampling location the tree adjacent to the soil pit and the two closest trees to this tree were selected (Fig. 6).

### Rainfall measurements

Rainfall was measured in gauges located near the centre of each open area. The rain gauges had a surface of 137 cm^2^, a capacity of 2-L, and were mounted at a height of 1.5 m. Rain was collected daily during the rainy season in 2008, 2009 and 2010.

Rainfall data was missing at the beginning of the rainy season (March-June 2008 and 2010), as the equipment was not yet installed. Simple linear regression was performed between 15 months of available data from Saponé and data from Ouagadougou meteorological station, the closest to the study area, for the same period. The regression model obtained (y = 11.3 + 1.1x; r^2^_adj_ = 0.8, p < 0.0001) was used to predict the mean monthly rainfall for the months with missing data.

### Soil water drainage measurements

Soil water drainage, the vertical flux of water within the vadose zone which can eventually become actual groundwater recharge when reaching the water table, was collected using passive capillary fiberglass wick lysimeters (adapted from those presented by Zhu *et al*.^56^). These passive capillary fiberglass wick lysimeters have a larger drainage collection efficiency than normal zero-tension pan lysimeters, which can approach 100%^56^.

Our lysimeters consisted of a 30 × 40 cm^2^ fiberglass plate with a slight slope which drained all collected water towards a hose connected to a 7-litre tank. The wick was an 8 mm diameter Thermo- E glass fiber twisted rope (*HKO Heat Protection* Group, Germany). The wicks were heated at 400 °C for 4 hours in order to remove any organic residues^57^. Two fiberglass ropes were inserted through the hose, leaving a piece of free rope on top of the plate. The lysimeters were filled to the top with 2 mm diameter sand. The remaining piece of fiberglass rope was separated into individual strands which were evenly arranged over the sand surface (Fig. 5e). This surface was then placed against the ceiling of a cavity dug in the wall of the soil pit (1.5 m beneath the soil surface and 50 cm from the pit wall) (Fig. 5d). Prior to the lysimeter installation the cavity ceiling was leveled to ensure contact between the lysimeter water collection surface and the lower surface of the undisturbed soil. A lysimeter of this type produces a hanging water column that exerts a negative pressure to the soil above the lysimeter^56^. The difference in elevation between the collection surface and the end of the wick was about 50 cm, which thus generated up to 50 cm of water tension on the soil^58^. Soil pits were covered with an unpainted galvanized iron sheet to reduce evaporation and avoid direct precipitation.

Water from the lysimeters was collected daily, in the early morning, during the rainy season in 2008, 2009 and 2010. Yearly soil draining water volumes were calculated by accumulating the daily collected draining soil water. Given the depth of the lysimeters installations we judged it a prerequisite that for meaningful readings the local groundwater must remain lower than 2 m depth. Therefore we only used data from days where the groundwater table was lower than 2 m depth in all the pits, which represented 85, 99 and 60% of the total annual rainfall for 2008, 2009 and 2010 respectively (Supplementary Table S1). The accumulated soil draining water volume for each year was scaled according to the total rainfall collected during the sampling period.

Three lysimeters malfunctioned during use, two in 2008 and one in 2009. These failures resulted from poor contact with the soil due to either flawed installation, erosion or disturbance by animals (the most likely being rodents or termites). Readings from these lysimeters have been removed from the data set.

### Sap flow measurements

Sap flow was measured using the heat ratio method (HRM)^59^, this is a modification of the Heat Pulse Velocity method^60^. Estimates of each Shea tree’s daily transpiration were obtained from accumulation of 24 hourly sap flow measurements from HRM30 heat ratio probes (ICT International, Armidale, Australia) (Fig. 5f), generated as in ref. 59. On each tree, four HRM30 sensors were installed at 1.30 m height along the compass directions East, West, North and South in order to account for circumferential variability. Each sensor was 35 mm long and contained three probes (a heater probe and two temperature probes, installed equidistantly upstream and downstream from the heater probe). Each temperature probe contained two thermocouples located at 7.5 and 22.5 mm. A heat pulse of 20 joules was automatically sent to the sensors every 10 minutes. The speed of propagation of the heat (*Vh*) was calculated according to ref. 60 every 10 minutes, and an average hourly value was automatically stored by an SL5 Smart Logger recorder (ICT International). Heat pulse velocity values were corrected for errors arising from probe misalignment and wounding following ref. 59. The corrected *Vh* values were then converted into sap velocity (*V*_*s*_) according to ref. 61. At the end of the measurement period, a wood core was extracted from each monitored tree using a Pressler increment borer in a random compass direction, and sapwood thickness was determined from this sample. We chose to sample sapwood thickness at a random compass direction to avoid any systematic errors due to potential patterns in sapwood depth around the tree. The cross-sectional sapwood area, estimated from the sapwood thickness assuming radial symmetry, was then divided into two concentric annuli delimited by the midpoint between the two measurement depths (7.5 mm and 22.5 mm). The sap flow corresponding to each annulus was estimated by multiplying the area of the annulus by *Vs* at the specific depth. The sap flow for the entire sap wood area was obtained by adding the values from the two concentric annuli. Finally, the tree transpiration rate was calculated by averaging the sap flow values from the four sensors installed in each tree.

Sap flow was measured simultaneously on three Shea trees per sampling location for a period of 10 to 50 days, after which the instruments were moved to another sampling location (see Supplementary Table S2 for details on the sampling periods at each location). In total 27 trees, corresponding to 9 sampling locations, were measured in both the rainy season and the dry season over the period July 2008–December 2010. The diameter at 1.3 m height (DBH) of the measured trees ranged from 19 to 90 cm, with a mean value of 55 cm, while their ground projected canopy area ranged from 28 to 190 m^2^, with a mean value of 93 m^2^. Wrong data, indicated by an error code, were removed. When hourly data were lacking, either due to wrong data or other technical problems, the gaps with missing data were replaced by the hourly average of the day if there were less than 5 gaps within that day. Otherwise, the data from that day was not used.

Sap velocity typically varies radially across the sapwood, with a peak generally occurring within the outermost portion of the sapwood depth, and from there decreasing towards the heartwood^62^,^63^,^64^,^65^. Failure to account for this radial variation in sap velocity can introduce significant errors when scaling up sap flow measurements from tree to stand^66^,^67^. When radial variation is not considered, single-point measurements at the outer sapwood can result in overestimation errors as large 300%^65^, or 154%^62^. Measuring sap velocity at two close sample points, similarly to what we did in this study, has been shown to systematically overestimate tree water use, with errors ranging between 14 and 81%^62^. To evaluate the effects of a potential overestimation of tree water use on groundwater recharge, we performed a sensitivity analysis assuming an overestimation error of 100%. We discuss the implications of this in more detail in the discussion section.

### Spatial model

We modelled annual groundwater recharge in Simile v5.9 software (Simulistics Ltd). The model consisted of a spatial grid of 1 ha (100 × 100 m), with cell size of 1 m^2^, and an overlying layer of the same extent which stored the X and Y coordinates of individual trees. Tree locations were randomly generated for each of the 100 simulations per input tree density (ranging from 1 to 70 trees ha^−1^). Soil water drainage at 1.5 m soil depth as percentage of annual rainfall was modelled in each of the grid cells as a function of the distance from its centre to the nearest tree stem according to the fitted relationship (Supplementary Fig. S5a, Fig.2). This relationship was determined from our 2009 data for two reasons: firstly the rainfall in this year (720 mm year^−1^) was very close to the long-term average (787 mm year^−1^); and secondly the sampling period included the least interrupted time series with 99% successful sampling of the annual rainfall (Supplementary Table S1). While in 2009 the lysimeters where installed and functioning during three months, in 2008 and 2010 the sampling period was reduced to two months and a half and one month and a half respectively. When comparing with the amount of time needed for water to reach the lysimeters furthest from trees (Fig. 4) it is evident that in the specific case of 2010, this meant that in these lysimeters much of the water did not have time to drain down to the lysimeters within this month and a half. Therefore, lysimeters located furthest from trees underestimated the accumulated drainage relative to the total rainfall during the sampling period as compared to data from 2008 and 2009. However, close to trees where percolation rates are faster, drainage was similar in all years–thus supporting that drainage is highest just beyond the tree crowns. To determine the effect of distance to tree on drainage, non-linear exponential regression was used for the interval of distances corresponding to sample points under the tree canopy and linear regression beyond the canopy edge (both performed using Minitab 16). Ordinary bootstrapping regression was also tested in the open areas using the *boot* function from the package *boot*^68^ in R^69^, using 1000 replicates. The obtained average regression coefficients were very similar to the ones obtained by ordinary least squares method, and therefore we only show these latter in the results. As a conservative choice, the linear relationship found was not extrapolated beyond our data but instead the value recorded in the largest open area (1.3% of annual rainfall, Fig. 2) was used as the minimum drainage beyond 36.8 m. Thus, depending on the distance to the nearest tree stem, the accumulated drainage at 1.5 m soil depth (% annual rainfall) was estimated according to the following three expressions:


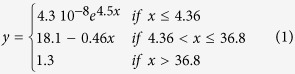


where *y* is the annual accumulated drainage at 1.5 m below the ground surface (% annual rainfall) and *x* is the distance to the nearest tree stem (m).

To calculate the groundwater recharge scenarios tree transpiration was subtracted from the drainage. Sap flow was derived from a per tree basis using a log-log regression (Minitab 16) between the natural log (Ln) of average daily sap flow from the 27 monitored trees and the Ln of their canopy area (y = 1.43 + 0.78x(SE 0.14); where y is Ln [sap flow] and x Ln [canopy area]; r^2^_adj_ = 0.53, p < 0.001). Subsequent conversion to express this relationship in natural units provides an exponential function (Supplementary Fig. S6).

We explored the influence of tree size with three simulated scenarios involving: small (40 m^2^ canopy area), average (67 m^2^) and large trees (130 m^2^). The predicted daily sap flow for each scenario, i.e. 76, 113, and 190 L tree^−1^day^−1^ respectively (Supplementary Fig. S6), was multiplied by tree density and by 365 to estimate annual transpiration losses.

The depth from which the transpired water was drawn by the trees in the study remains uncertain. The vertical profile of this extraction process is hard to assess and remains undetermined. To address this uncertainty we modelled annual groundwater recharge under 5 different model scenarios covering the full range of possibilities. In the first model we considered that trees extract 100% of transpired water from below 1.5 m soil depth. The remaining models assumed 75%, 50%, 25% and 0%. Annual groundwater recharge was subsequently obtained for each model by extracting the adjusted volume of transpired water from the annual accumulated drainage at 1.5 m soil depth. Each drainage model was run 100 times for each of the 30 different tree densities ranging from 1 tree ha^−1^ to 70 trees ha^−1^. All trees positions were randomized for each of the 3000 simulations.

Furthermore, in order to evaluate the influence of how trees are distributed in space we ran seven simulations (1, 4, 9, 16, 25, 36 and 49 trees ha^−1^) with a regularly spaced “square” distribution of trees. We considered average tree size (67 m^2^ canopy area) and 100%, 75%, 50%, 25% and 0% assumptions for extraction of transpired water.

Our original model assumed that drainage at 1.5 m soil depth and groundwater recharge are influenced only by the distance to the nearest tree stem. To explore the influence of any possible additive effect in which groundwater recharge is influenced by multiple neighboring trees we modified our initial *nearest-neighbour* model. In our *additive* model, we assumed that drainage at 1.5 m was highest at the canopy edge, 4.36 m from the tree, and then decreased linearly down to 1.3% of annual rainfall at 36.8 m from the tree. We assumed drainage was zero under tree canopies, and 1.3% beyond 36.8 m away from any tree. The effect of trees on drainage was then summed when the ranges between 4.36 and 36.8 m around any trees were overlapping. We assumed that the per-hectare mean drainage value from our original model was valid for a tree density of 20 trees ha^−1^, since this is the average tree density in our study area. We calibrated the effect of the individual trees in our *additive* model so that the original and *additive* models gave equal results at a tree density of 20 trees ha^−1^. The drainage effect from each tree in these circumstances was y = 3.5 − 0.06x (where x is the distance to the tree in m) between 4.36 and 36.8 m. The model was run 100 times for each of the 30 input tree densities ranging from 1 tree ha^−1^ to 70 trees ha^−1^ assuming random distribution of trees, average tree size (67 m^2^ canopy area), and 50% water uptake below 1.5 m soil depth (for example outputs see Supplementary Fig. S5b).

## Additional Information

**How to cite this article**: Ilstedt, U. *et al*. Intermediate tree cover can maximize groundwater recharge in the seasonally dry tropics. *Sci. Rep.* **6**, 21930; doi: 10.1038/srep21930 (2016).

## Supplementary Material

Supplementary Information

## Figures and Tables

**Figure 1:**
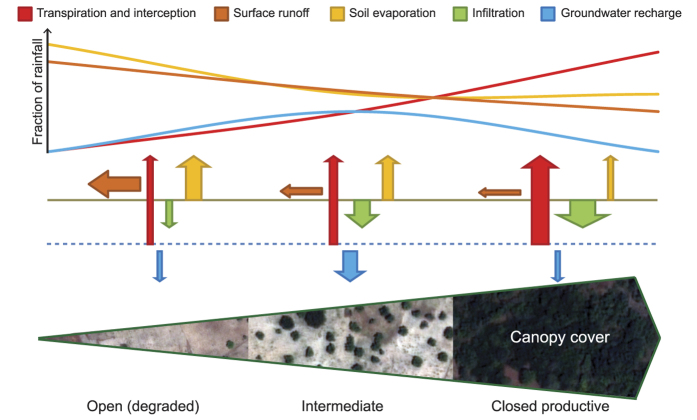
Conceptual water budget of the *optimum tree cover theory.* Optimum groundwater recharge occurs at intermediate tree cover in seasonally dry tropical areas. Without trees, surface runoff and soil evaporation are high, leading to low groundwater recharge despite low transpiration. In closed productive forests, despite low surface runoff and soil evaporation, total transpiration and interception are high, again leading to low groundwater recharge. At an intermediate canopy cover, low surface runoff and evaporation as well as intermediate transpiration optimize groundwater recharge. The pan-sharpened satellite images were created from a WorldView-2 image from 21 October 2012 using ERDAS Imagine 2013 software (http://www.hexagongeospatial.com/products/producer-suite/erdas-imagine).

**Table 1:** Number of sampling locations (total, large open areas and smaller open areas) for each year.

Year	Sampling location		
	Total	Large open areas	Small open areas
2008	6	3	3
2009	8	4	4
2010	9	4	5
