# Supplementary material for: Intermediate tree cover can maximize groundwater recharge in the seasonally dry tropics
Source: Sci Rep. 2016 Feb 24;6:21930. doi: 10.1038/srep21930 (PMC4764941; doi:10.1038/srep21930)
Supplement: Supplementary Information [file srep21930-s1.pdf]

Supplementary information

Title: **Intermediate tree cover can maximize groundwater recharge in the seasonally dry tropics**

Authors: U. Ilstedt, A. Bargués Tobella, H.R. Bazié, J. Bayala, E. Verbeeten, G. Nyberg, J. Sanou, L. Benegas, D. Murdiyarso, H. Laudon, D. Sheil, A. Malmer

1. Supplementary Figures and Legends S1-S9

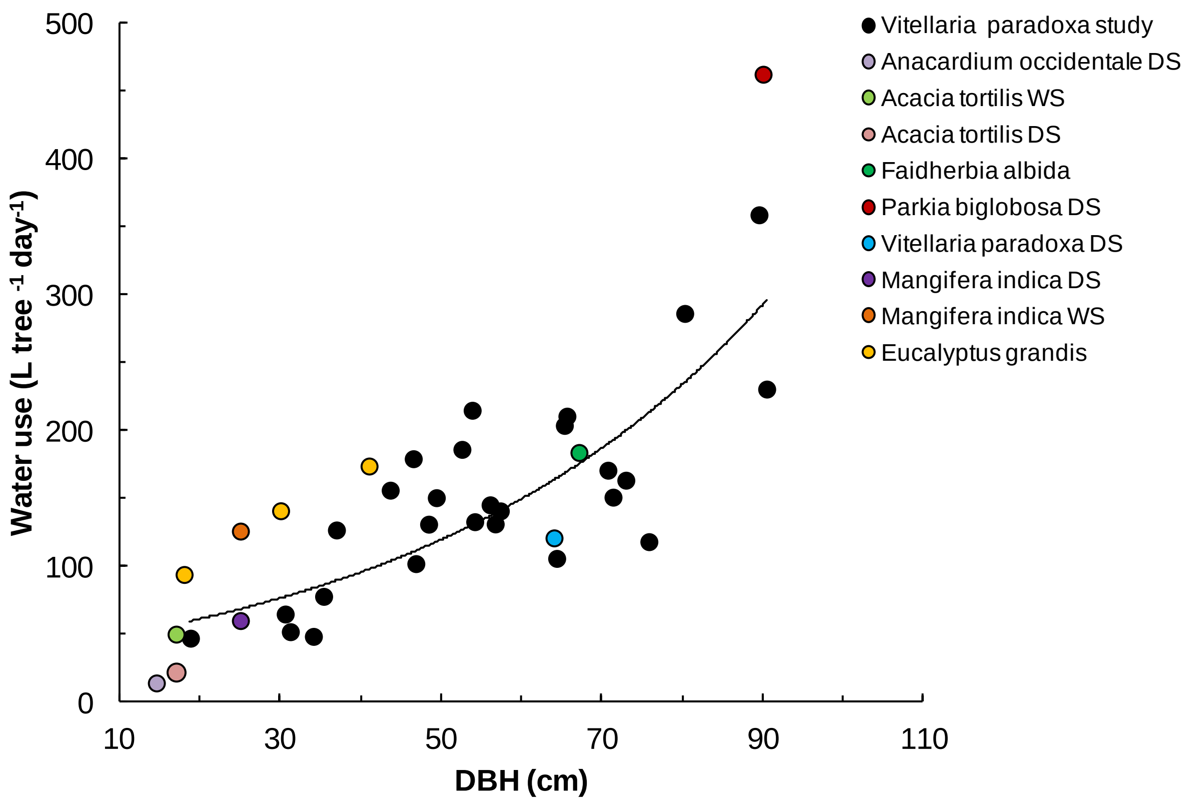

Supplementary Figure S1. Relationship between diameter at 1.3 m height (DBH) and daily water use for different tree species that can be found in agroforestry parklands in the semi-arid tropics. The black points correspond to data from the 27 Shea trees (*Vitellaria paradoxa* C.F. Gaertn.) monitored in this study and the black line shows the relationship found between

DBH and water use for these trees (LN [water use]=3.66+0.02 DBH, $r^2_{adj}=0.62$, $p<0.0001$). The colored dots show daily water use values for other tree species common in semi-arid tropical parklands measured during the dry season (DS) and/or the wet season (WS) in different areas: *Anacardium occidentale* L. plantation in Ghana[1]; *Acacia tortilis* (Forsk.) Hayne *ssp. raddiana* (Savi) Brenan var. *raddiana*, grazing land, Senegal[2]; *Faidherbia albida* (Del.) A. Chev. parkland, Burkina Faso[3]; *Parkia biglobosa* (Jacq.) Benth and *Vitellaria paradoxa* C.F. Gaertn. parkland, Burkina Faso[4]; *Mangifera indica* L. orchard, Australia[5] and *Eucalyptus grandis* W. Hill ex Maid plantations, South Africa[6].

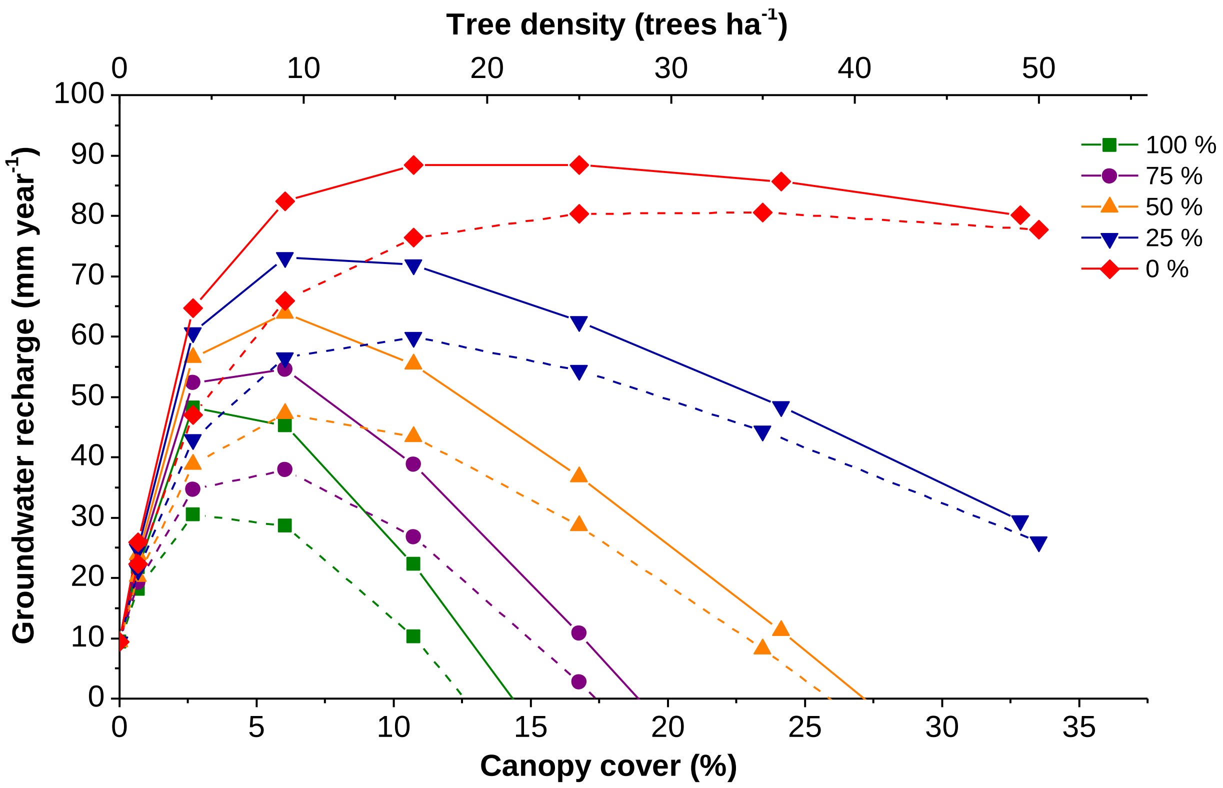

Supplementary Figure S2. Comparison of the effect of random and even tree distribution on groundwater recharge (mm year^{-1}). Spatial simulations based on sap flow measurements and the observed relationship between drainage below 1.5 m depth in 2009 and distance to the nearest tree. The dashed lines depict mean values for the 100 simulations for each tree density where trees have been positioned randomly while the solid lines represent the single outcome for a squared regularly spaced distribution of trees. The colors/symbols represent different assumptions regarding the proportion (0%, 25%, 50%, 75%, 100%) of transpired water extracted below 1.5 m depth. Average tree size (67 m^2 canopy area) is assumed for all the simulations.

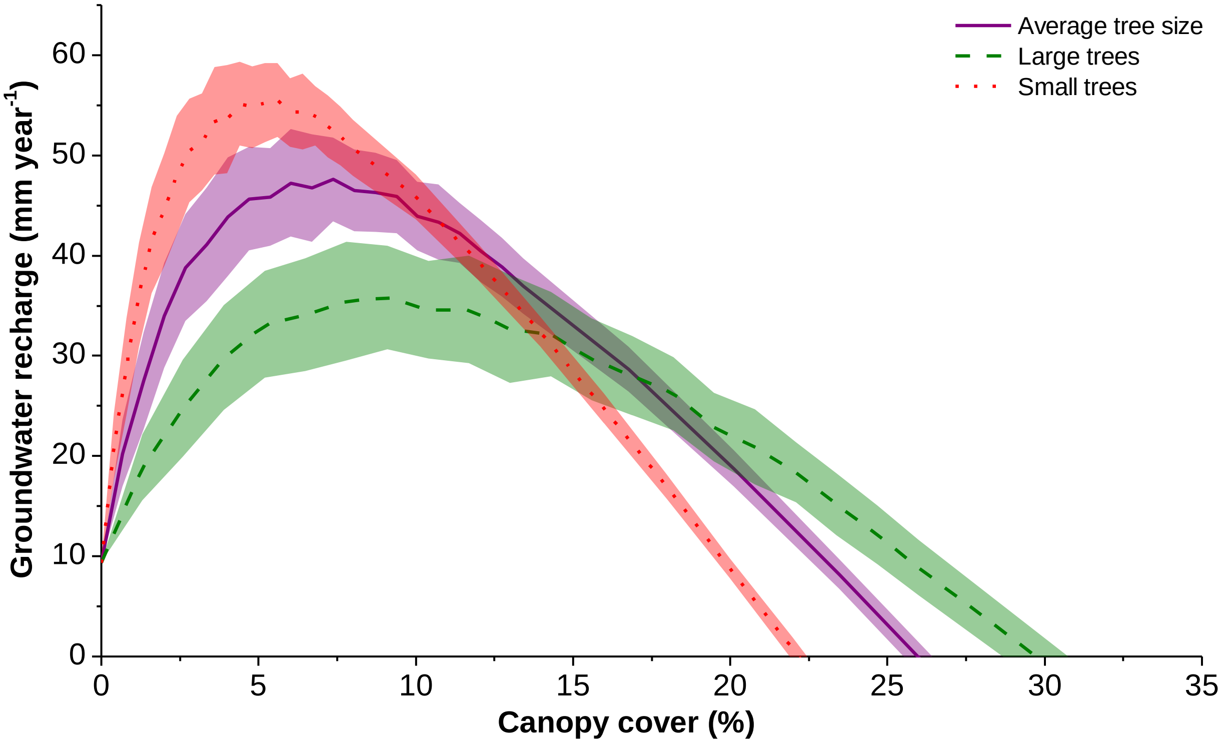

Supplementary Figure S3. Comparison of the effect of tree size on groundwater recharge (mm year^{-1}). Spatial simulations based on sap flow measurements and the observed relationship between drainage below 1.5 m depth in 2009 and distance to the nearest tree. For each canopy cover (%) the mean and ±SD of 100 simulations per tree density are shown. The effect of tree size is depicted by the different colors: the red (dotted), purple (solid) and green (dashed) lines are for trees with small, average and large canopy areas respectively (40 m^2, 67 m^2 and 130 m^2). In all these simulations 50 % of transpired water is assumed to originate from at least 1.5 m belowground.

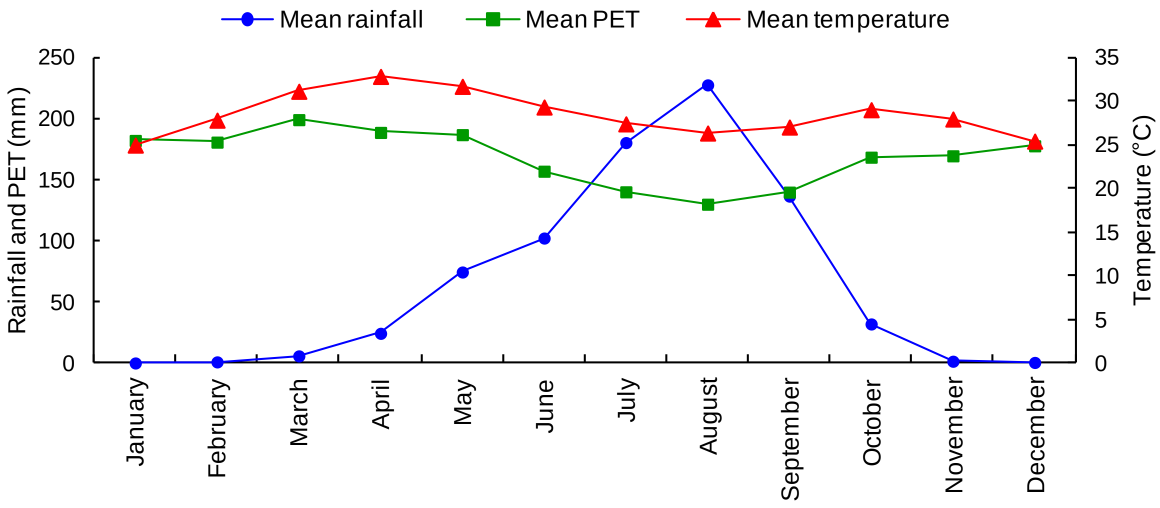

Supplementary Figure S4. Meteorological data. Mean monthly rainfall (1952-2010), potential evapotranspiration (PET; 1974-2003) and temperature (1952-2008) recorded at Ouagadougou Meteorological Station (Source: Direction de la Météorologie du Burkina Faso).

a

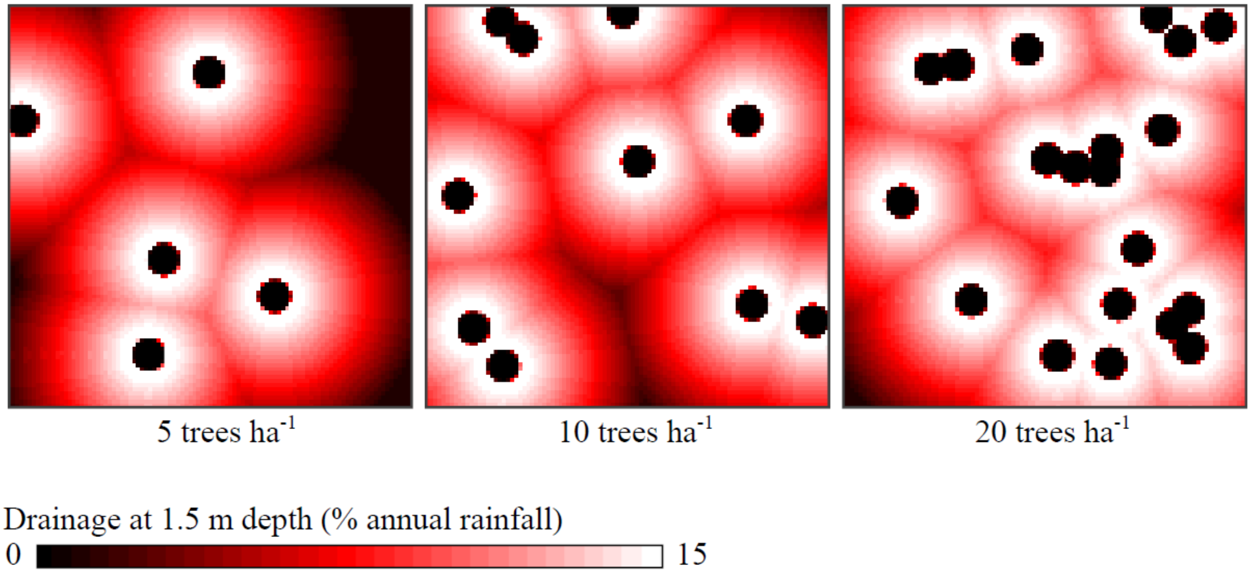

b

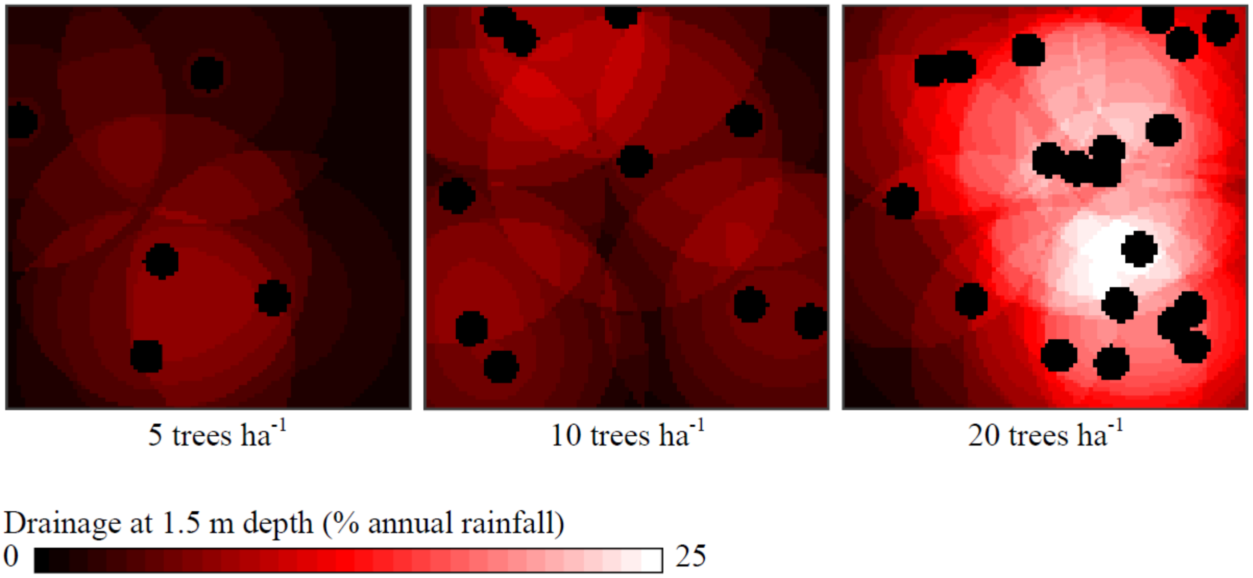

Supplementary Figure S5. Examples of the spatial model output. Examples of one hectare spatial simulations of soil water drainage at 1.5 m depth expressed as the percentage of annual rainfall for tree densities of 5, 10 and 20 trees ha^{-1}, using **(a)** a *nearest-neighbour* model and **(b)** an *additive* model for the influence of trees on drainage.

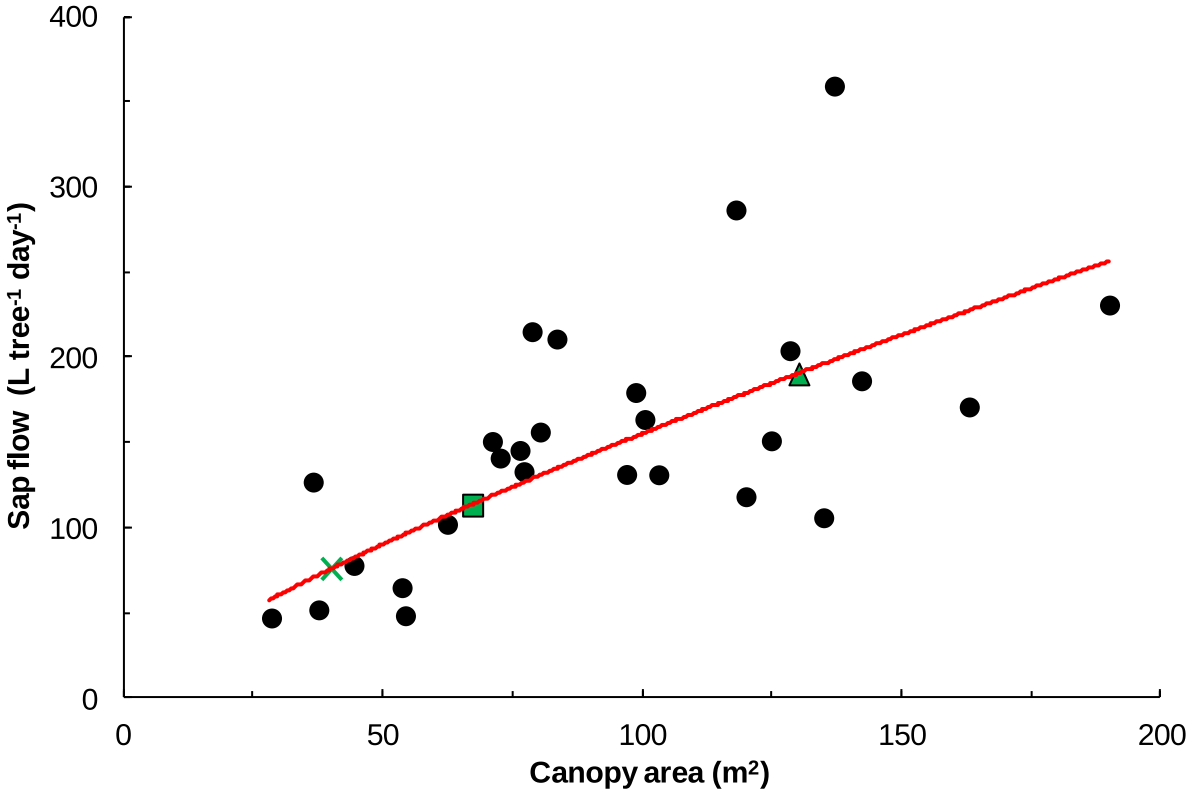

Supplementary Figure S6. Scatter plot relating canopy area and recorded daily sap flow for 27 trees (black dots). The red line corresponds to the exponential regression function obtained by using the antilogarithms of the logarithmic function (Sap flow=4.19 canopy area $^{0.78}$, r^2_{adj}=0.53, p<0.001). The green square indicates the predicted sap flow (113 L tree^{-1} day^{-1}) for the tree with average crown area (67 m^2). The green cross and the green triangle indicate the predicted sap flow (76 and 190 L tree^{-1} day^{-1}) for trees with small and large crown areas respectively (40 m^2 and 130 m^2).

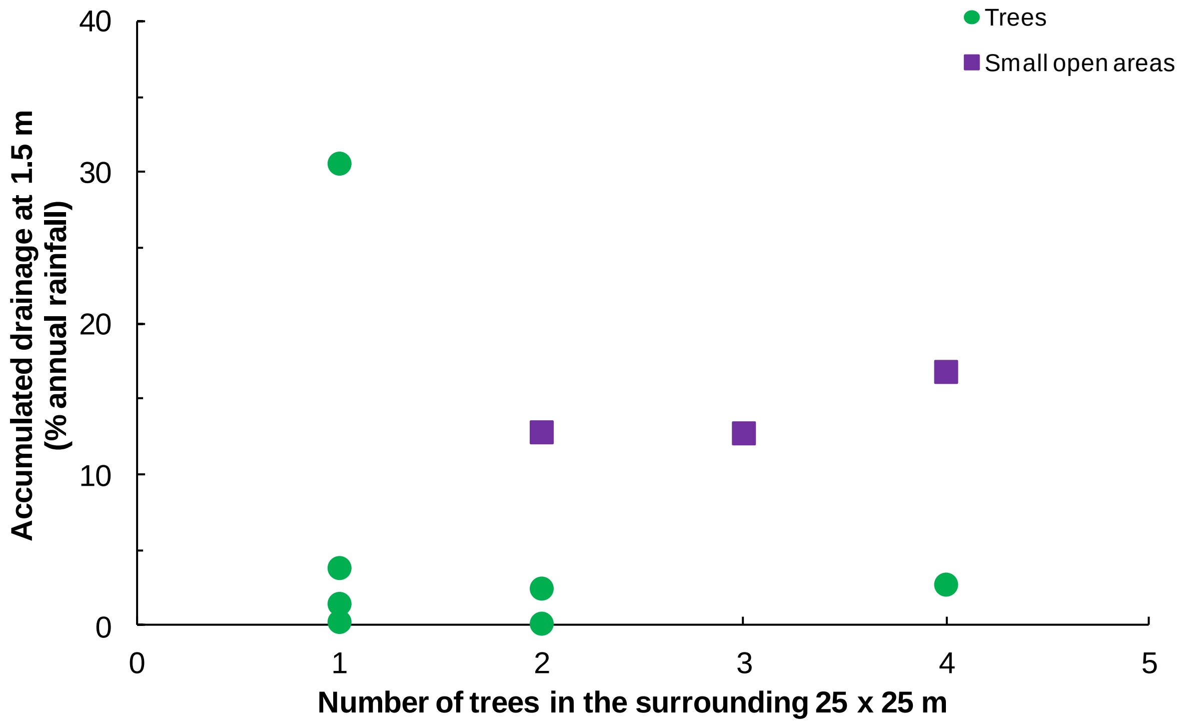

Supplementary Figure S7. Scatter plot of the number of trees in the 25 x 25 m area surrounding a soil pit where soil water drainage was collected at 1.5 m belowground versus the accumulated drainage; based on 2009 data from soil pits located at the center of small open areas (purple squares) and under the tree canopy (green circles).

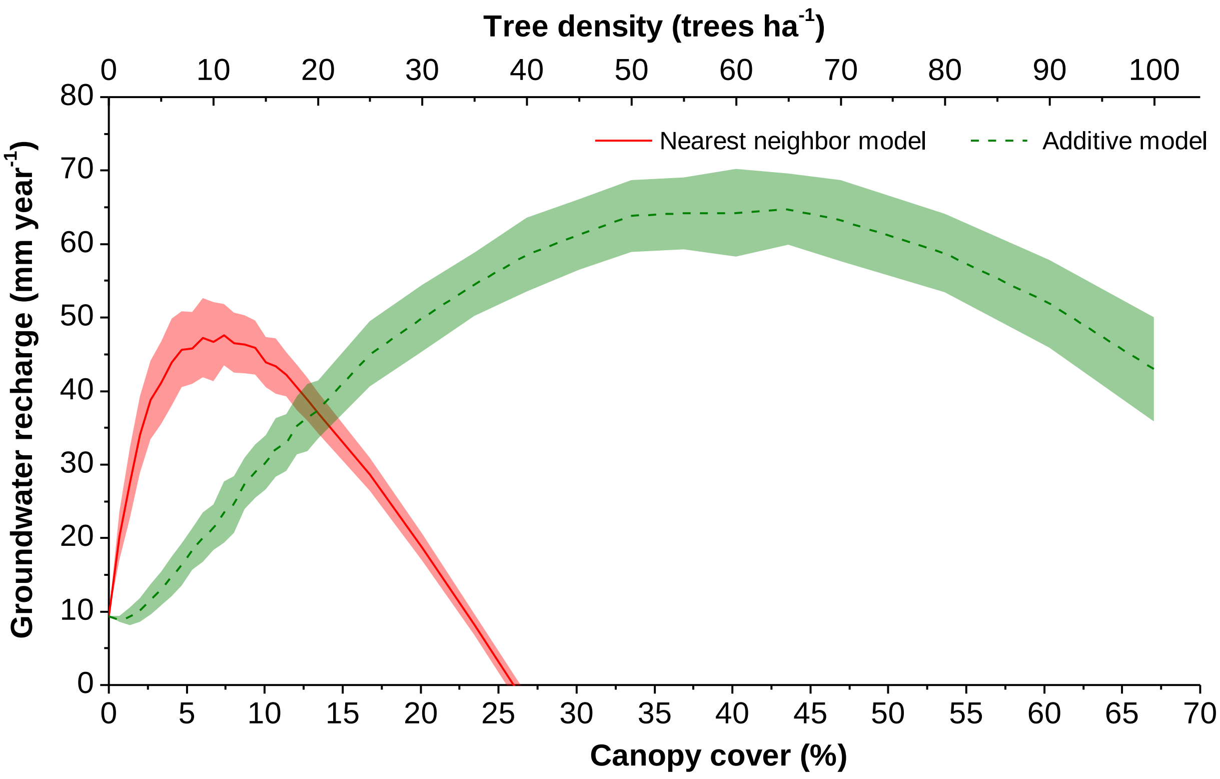

Supplementary Figure S8. Comparison of the effect of an *additive* model versus the *nearest-neighbour* model on groundwater recharge (mm year^{-1}). For each canopy cover (%) the mean and ±SD of 100 simulations per tree density are shown. The original *nearest-neighbour* model, where no additive effect was accounted for, is depicted by the red continuous line while the more complex *additive* model that allows for the effect of multiple trees is depicted by the green dashed line. In all these simulations it is assumed that 50 % of transpired water is extracted from at least 1.5 m belowground and that trees are average sized (67 m^2 canopy area).

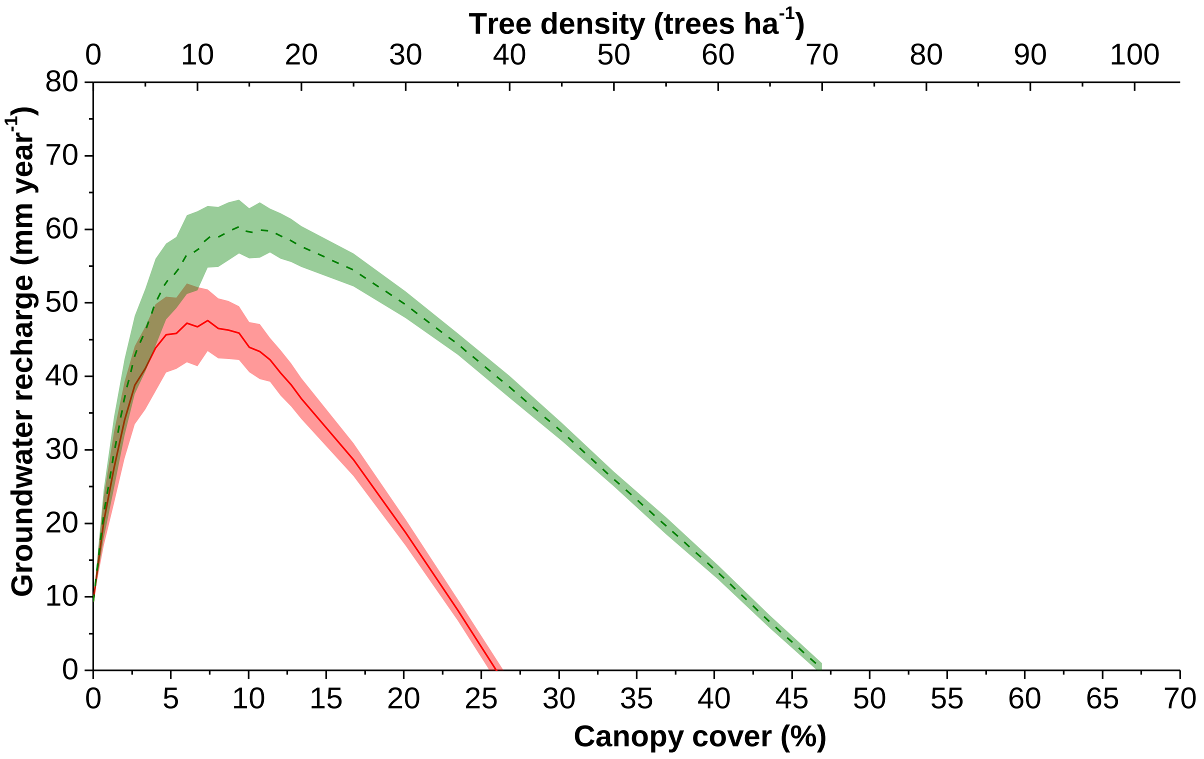

Supplementary Figure S9. Effect on groundwater recharge estimates (mm year^{-1}) from potential overestimation of tree water use due to radial variation in sap velocity. For each canopy cover (%) the mean and ±SD of 100 simulations per tree density are shown. The original model is depicted by the red continuous line while the green dashed line depicts the scenario where a potential overestimation error of 100% in the original estimates of tree water use has been accounted for. In all these simulations it is assumed that 50 % of transpired water is extracted from at least 1.5 m belowground and that trees are average sized (67 m^2 canopy area).

- **Supplementary Tables S1 and S2**

Supplementary Table S1. Annual rainfall by year, sampling periods (dates where the groundwater table was above 2 m depth have been excluded), and accumulated rainfall during the sampling periods in Saponé

Year	Annual rainfall (mm)	Sampling period	Rainfall in sampling period (mm)
2008	836	29 June -15 Sept.	710
2009	720	9 July - 13 Oct.*	712
2010	1175	1 July - 13 Aug.	702

*****27 Aug. and 2 Sept. excluded

Supplementary Table S2. **Sap flow measurement periods and corresponding sampling locations. Sampling locations corresponding to small open areas are indicated by an S while those corresponding to large open areas are indicated by an L. The sampling periods with wrong data are also shown.**

Measurement Period	Sampling location	Observations
14 July - 25 Aug. 2008	L2	Wrong data
25 Aug.- 09 Sept. 2008	L2	
12 Sept.- 26 Sept. 2008	L3	
01 Oct. - 11 Oct. 2008	S2	
11 Oct. - 24 Oct. 2008	L1	
30 Oct. - 18 Nov. 2008	S3	
19 Nov. 2008 -21. May 2009		Logger did not work
22 May - 16 June 2009	S1	
16 June - 03 July 2009	S3	
03 July - 21 July 2009	L1	
24 July - 14 Aug. 2009	S2	
14 Aug. - 29 Sept. 2009	L2	
15 Oct. - 11 Nov. 2009	L3	
11 Nov. - 03 Dec. 2009	L3	Wrong data
03 Dec. 2009 - 14 Jan. 2010	L4	
14 Jan. - 18 Feb. 2010	S4	
04 Mar. - 08 Apr. 2010	S1	
08 Apr. - 05 May 2010	S3	
31 May - 06 July 2010	S3	
21 July - 03 Aug. 2010	L1	
03 Aug. - 24 Aug. 2010	L4	
24 Aug. - 16 Sept. 2010	S4	
17 Sept. - 10 Oct. 2010	S2	Wrong data
10 Oct. - 09 Nov. 2010	L2	
23 Nov. - 09 Dec 2010	L3	
09 Dec. - 31 Dec. 2010	S5	

- **Supplementary Discussion**

Upscaling of point measurements to the plot level can give rise to potential sources of error. Here, we would like to discuss a couple of such sources of error in order to evaluate the validity of our main conclusions.

A first potential source of error can originate when scaling up soil water drainage measured at 1.5 m soil depth to the plot level. In the simplest *nearest-neighbour* version of our spatial model we consider that drainage at 1.5 m depends only on the nearest tree to a given point. We believe this is a reasonable approximation in the context we are considering since our data does not indicate any additive effect in which the model can be influenced by the overall number of trees (Supplementary Fig. S7). We recognize that our spatial replication is limited to detect such effect and that this could be more pronounced in other situations. Therefore, we assessed the implications of an *additive* version of the model in which drainage at 1.5 m depends not only on the nearest tree to a given point but on distance to all trees. These simulations also yield unimodal patterns but the maximum ground water recharge is 17 mm year^{-1} higher and broader and occurs at higher tree densities (Supplementary Fig. S8). Notably the optimum canopy cover increased from 7% to 43% in the *additive* model, assuming 50% water uptake below 1.5 m. Whether the *nearest-neighbour* model, the *additive* model, or some combination of these, is the best reflection of reality, and whatever depth the trees draw most of their moisture from, our main conclusions remain unaffected: there will be a non-zero tree cover value that maximizes ground water recharge.

The upscaling of sap flow from point measurements to the whole-tree level, and from the individual tree to the stand level are additional potential sources of error. Our sensitivity analysis

on the effect of potential overestimation errors in tree water use derived from not adequately accounting for radial variation in sap velocity shows that assuming a potential overestimation error of 100% in our original estimates results in more groundwater recharge under the optimal canopy cover, and also in an increase in the tree density at which this optimum occurs (Supplementary Fig. S9). Upscaling from the tree to the stand level also can lead to further sources of error. When scaling up sap flow it is important to select a representative sample of trees to monitor, whose range in size covers the actual range in size of the stand trees[7], and we have done that. Stand transpiration under the different tree density scenarios was calculated based on the relationship found between canopy area and daily sap flow per individual tree, and assuming that all trees in the stand had the same size (average, large and small trees scenarios were considered). We admit that this assumption is not fully realistic. Indeed, stand transpiration will vary according to spatial variability in tree water use due to a number of factors such as tree age, density and variation in soil moisture and depth[8]. Most likely tree transpiration will not always increase linearly with increasing tree density as we have assumed, but the increase per added tree will be less once tree root systems and canopies start overlapping due to competition and reduced leaf area per tree. All the above mentioned potential sources of error linked to sap flow upscaling most likely lead to overestimations of tree water use in our models. Hence, even if these sources of error might affect the specific value of canopy cover at which groundwater recharge is maximized, our main conclusion that an intermediate tree cover for optimal groundwater recharge exists will not change. Further research on the relationships between stand structure and water use is needed to be able to improve estimates of groundwater recharge from various types of tree cover.

- **Supplementary Information References**

1 Oguntunde, P. G. Water use pattern and canopy processes of cashew trees during a drying period in West Africa. *Int. J. Plant Prod.* 1, 85-98 (2007).

2 Do, F. C. *et al.* Stable annual pattern of water use by Acacia tortilis in Sahelian Africa. *Tree Physiol.* 28, 95-104 (2008).

3 Roupsard, O. *et al.* Reverse phenology and dry-season water uptake by Faidherbia albida (Del.) A. Chev. in an agroforestry parkland of Sudanese west Africa. *Funct. Ecol.* 13, 460-472 (1999).

4 Bayala, J., Heng, L. K., van Noordwijk, M. & Ouedraogo, S. J. Hydraulic redistribution study in two native tree species of agroforestry parklands of West African dry savanna. *Acta Oecol.* 34, 370-378 (2008).

5 Lu, P. & Chacko, E. K. in *5th International Mango Symposium, Vols 1 and 2 Acta Horticulturae* (eds U. Lavi *et al.*) 339-350 (1997).

6 Wullschleger, S. D., Meinzer, F. C. & Vertessy, R. A. A review of whole-plant water use studies in trees. *Tree Physiol.* 18, 499-512 (1998).

7 Čermák, J., Kučera, J. & Nadezhdina, N. Sap flow measurements with some thermodynamic methods, flow integration within trees and scaling up from sample trees to entire forest stands. *Trees* 18, 529-546 (2004).

8 Asbjornsen, H. *et al.* Ecohydrological advances and applications in plant–water relations research: a review. *J. Plant Ecol.* **4**, 3-22 (2011).
